# Description of *Uraechanigromaculata* sp. n. (Coleoptera, Cerambycidae, Lamiinae) from Guizhou, China

**DOI:** 10.3897/BDJ.11.e104253

**Published:** 2023-05-18

**Authors:** Sheng Liang, Liang Guo, Weicheng Yang, Shulin Yang

**Affiliations:** 1 Xishui National Nature Reserve, Xishui, China Xishui National Nature Reserve Xishui China; 2 School of Life Sciences, Guizhou Normal University, Guiyang, China School of Life Sciences, Guizhou Normal University Guiyang China

**Keywords:** Longhorn beetles, taxonomy, Xishui, Natural Nature Reserve

## Abstract

**Background:**

The genus *Uraecha* Thomson, 1864 is an Asiatic genus. In China, *Uraechaangusta* (Pascoe, 1856) is the most common species and it is widely distributed in the southern part of the country. Two species, *U.angusta* and *Uraechaobliquefasciata* Chiang, 1951, are distributed in Guizhou Province of China. The type locality of the latter is Guiyang, the capital of Guizhou Province.

**New information:**

*Uraechanigromaculata* **sp. n.** is described and illustrated. A diagnosis is presented to distinguish this species from its close relatives. It is the third species of the genus *Uraecha* reported from Guizhou Province.

## Introduction

The genus *Uraecha* Thomson, 1864 currently consists of 16 taxa which are exclusively distributed in Asia. Eight species of the genus have so far been recorded from China; two of them, *Uraechaangusta* (Pascoe 1856) and *Uraechaobliquefasciata* (Chiang 1951), are distributed in Guizhou Province (Wang and Chiang 2000, Tavakilian and Chevillotte 2022, Lin and Ge 2017). Based on the specimens collected from the Xishui National Nature Reserve (Xishui, Guizhou, China), we identified and described a new species, *Uraechanigromaculata* **sp. n.**, in this article.

## Materials and methods

Individuals used in this study were collected using flight interception traps. Morphology was examined using an AmScope SM-4TZ stereomicroscope. Images of habitus were taken with a Canon EOS 6D digital camera fitted with a Carl Zeiss Milvus 100 mm lens. Male terminalia were photographed using a digital camera connected to the AmScope SM-4TZ stereomicroscope. Two specimens of *U.angusta* (the closest relative) were examined for comparison. These materials were collected from the same localities as the new species during the same period. All materials are deposited in the collection of the School of Life Sciences, Guizhou Normal University, Gui’an New Area, China (GZNULS).

## Taxon treatments

### Uraecha nigromaculata sp. n.

C5C65459-5455-5A6C-A10A-AE5FD766E54E

412B5A4E-654D-44CA-939D-0B901E9433F7

#### Materials

- **Type status:** Holotype. **Occurrence:** recordedBy: Shulin Yang and Run Shi; individualCount: 1; sex: male; lifeStage: adult; occurrenceID: B8DC6079-27EA-5811-B8F4-4D086DBEE615; **Taxon:** scientificName: Uraechanigromaculata; **Location:** country: China; stateProvince: Guizhou; county: Xishui; locality: Tiantanggou, Sanchahe; verbatimCoordinates: 106°23.662'E, 28°31.992'N; **Identification:** dateIdentified: 2022; **Event:** samplingProtocol: flight interception trap; year: 2022; month: 10; day: 17
- **Type status:** Paratype. **Occurrence:** recordedBy: Shulin Yang and Run Shi; individualCount: 1; sex: female; lifeStage: adult; occurrenceID: 7DC890E5-9AB6-545B-B9AA-E092F93C1E46; **Taxon:** scientificName: Uraechanigromaculata; **Location:** country: China; stateProvince: Guizhou; county: Xishui; locality: Hongyangou, Sanchahe; verbatimCoordinates: 106°23.613’ E, 28°29.590’N; **Identification:** dateIdentified: 2022; **Event:** samplingProtocol: flight interception trap; year: 2022; month: 10; day: 17
- **Type status:** Paratype. **Occurrence:** recordedBy: Shulin Yang and Run Shi; individualCount: 1; sex: male; lifeStage: adult; occurrenceID: 278D8D11-D8D2-59FD-8AB3-A83E2EC0BB40; **Taxon:** scientificName: Uraechanigromaculata; **Location:** country: China; stateProvince: Guizhou; county: Xishui; locality: Hongyangou, Sanchahe; verbatimCoordinates: 106°23.613’ E, 28°29.590’N; **Identification:** dateIdentified: 2022; **Event:** samplingProtocol: flight interception trap; year: 2022; month: 10; day: 17
- **Type status:** Paratype. **Occurrence:** recordedBy: Shulin Yang and Run Shi; individualCount: 1; sex: male; lifeStage: adult; occurrenceID: 1FF5AD80-2484-5CDE-9FFB-786FB74C4828; **Taxon:** scientificName: Uraechanigromaculata; **Location:** country: China; stateProvince: Guizhou; county: Xishui; locality: Hongyangou, Sanchahe; verbatimCoordinates: 106°23.613’ E, 28°29.590’N; **Identification:** dateIdentified: 2022; **Event:** samplingProtocol: flight interception trap; year: 2022; month: 10; day: 17
- **Type status:** Paratype. **Occurrence:** recordedBy: Shulin Yang and Run Shi; individualCount: 1; sex: female; lifeStage: adult; occurrenceID: 2BD1E48E-6FAF-5BDB-9606-EA1A4AEDEAC7; **Taxon:** scientificName: Uraechanigromaculata; **Location:** country: China; stateProvince: Guizhou; county: Xishui; locality: Dabaitang; verbatimCoordinates: 106°16.437’ E, 28°26.138’N; **Identification:** dateIdentified: 2022; **Event:** samplingProtocol: flight interception trap; year: 2022; month: 5; day: 24
- **Type status:** Paratype. **Occurrence:** recordedBy: Shulin Yang and Run Shi; individualCount: 1; sex: female; lifeStage: adult; occurrenceID: 0C1980A1-4E9B-5990-AE6E-D19C708D9E92; **Taxon:** scientificName: Uraechanigromaculata; **Location:** country: China; stateProvince: Guizhou; county: Xishui; locality: Xiaoba; verbatimCoordinates: 105°56.083’ E, 28°16.616’N; **Identification:** dateIdentified: 2022; **Event:** samplingProtocol: flight interception trap; year: 2022; month: 6; day: 15

#### Description

**Figure 1.**
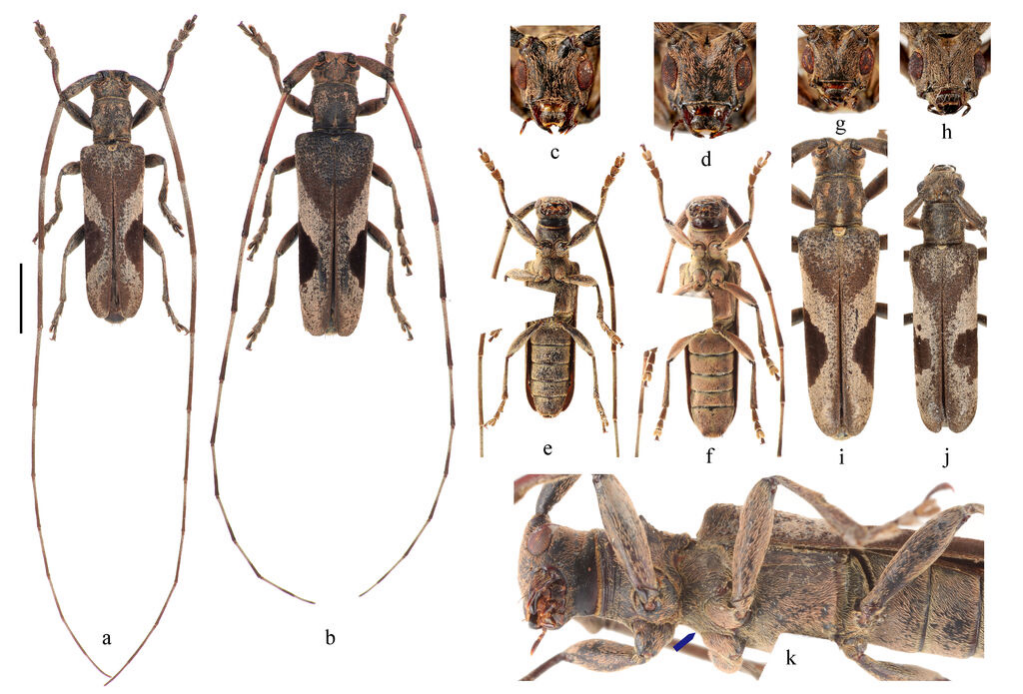
Habitus of *Uraechanigromaculata* sp. n. **a, c, e, k** holotype; **b, d, f, g, h, i, j** paratypes. (**a, c, e, g, i, k** male; **b, d, f, h, j** female; **c, d, g, h** head, front view; **a, b, i, j** dorsal view; **e, f** ventral view; **k** lateral view. Scale bar, **a, b** 5 mm; rest of the pictures not to scale.).

**Figure 2.**
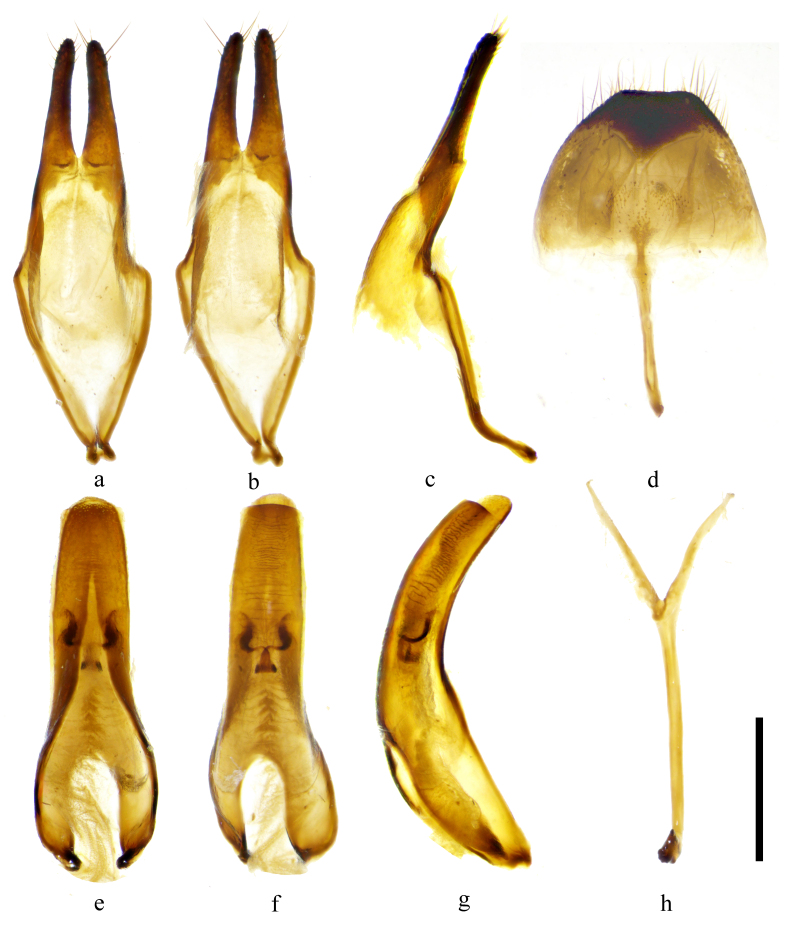
Male terminalia of *Uraechanigromaculata* sp. n. **a–c** parameres; **d** tergite VIII and sternites VIII; **e–g** median lobe; **h** spiculum gastrale. (**a, d, e, h** ventral view; **b, f** dorsal view; **c, g** lateral view). Scale bar 0.5 mm.

**Male**. **Body**: length: 16.8–23.1 mm, humeral width: 4.7–6.6 mm (holotype and 2 paratypes). **Holotype** (Fig. 1a, c, e, k), body length: 17.9 mm, humeral width: 4.7 mm, black, covered with greyish-yellow to light orange pubescence. **Head**: black, densely and finely punctured, scattered with several coarse punctures, dispersed with dense pubescence clusters; frons with a glabrous median carina which extends from base of clypeus through vertex (Fig. 1c, d, g, h). Antennae exceed apex of elytra by six antennomeres; antennal tubercles strongly raised, densely covered with long pubescence; scape and pedicel black, punctures coarser on pedicel than punctures on scape; cicatrix complete, narrow; rest of the antennomeres generally reddish-brown at basal half and darker at apical half, pubescence strong and thick at basal half, gradually finer and sparser towards apex which presents an annulate pattern. Eyes deeply emarginated; lower lobe twice as high as gena, frons’ widest width between lower lobes of eyes two times more than the width of lower lobe of eyes. **Thorax**: Pronotum black and covered with sparse granules, disc slightly raised in the middle and depressed at both sides behind lateral spines. Five longitudinal light orange stripes on pronotum (Fig. 1i), two stripes at the middle of each apical half, two stripes starting from the base of lateral spines and extending posteriorly to the base of pronotum, the fifth stripe at the middle of the base of pronotum, as long as 1/4 length of pronotum. Lateral spines weak, blunt, slightly posteriorly and upwardly curved. Scutellum covered with dense yellowish pubescence, apex rounded. Mesosternal process even, without projection (Fig. 1k). **Elytra**: subparallel laterally; with two large pubescent patches on each elytron, a light brown patch at base and a large subtriangular and semicircle-shaped dark brown patch behind the middle; hind of the basal patch extends obliquely from humerus towards suture and reaches suture at nearly half of elytral length; rest of the elytron covered with greyish-yellow pubescence with small brown pubescent patches dispersed, except for a short narrow brown band along suture at apical 1/5; irregular coarse granules intermingled with light orange to greyish pubescence clusters at basal 1/5, followed by punctures which are gradually smaller and sparser towards apex and generally not extending beyond middle, these punctures are denser along suture; apical subtruncate (Fig. 1a, b). Ventral surface with uniform greyish-yellow to light orange pubescence (Fig. 1e, f). **Male terminalia**: Tergite VIII (Fig. 2d) gradually constricted towards apex, truncated at apex, with sparse long setae at apex, setae in the middle of apex sparser than setae on sides of apex; sternite VIII (Fig. 2d) transverse, with sparse short setae at the middle. Lateral lobe slender, about 1/3 length of tegmen, widest at base, constricted to about 2/3 width of base at basal 1/3, then gradually tapering to apex, with sparse short setae and several long setae at apical 1/3 (Fig. 2a–c). Median lobe moderately curved, as long as tegmen, median strut shorter than half of median lobe, ventral plate rounded at apex (Fig. 2e–g). Length of spiculum gastrale (Fig. 2h) about twice the length of spiculum relictum (Fig. 2d).

**Female**. Body length: 16.3–20.5 mm, humeral width: 4.6–6.7 mm (3 paratypes, Fig. 1b, d, f, h, j). Similar to male, except antennae shorter, exceeding apex of elytra by five antennomeres.

#### Diagnosis

**Figure 3.**
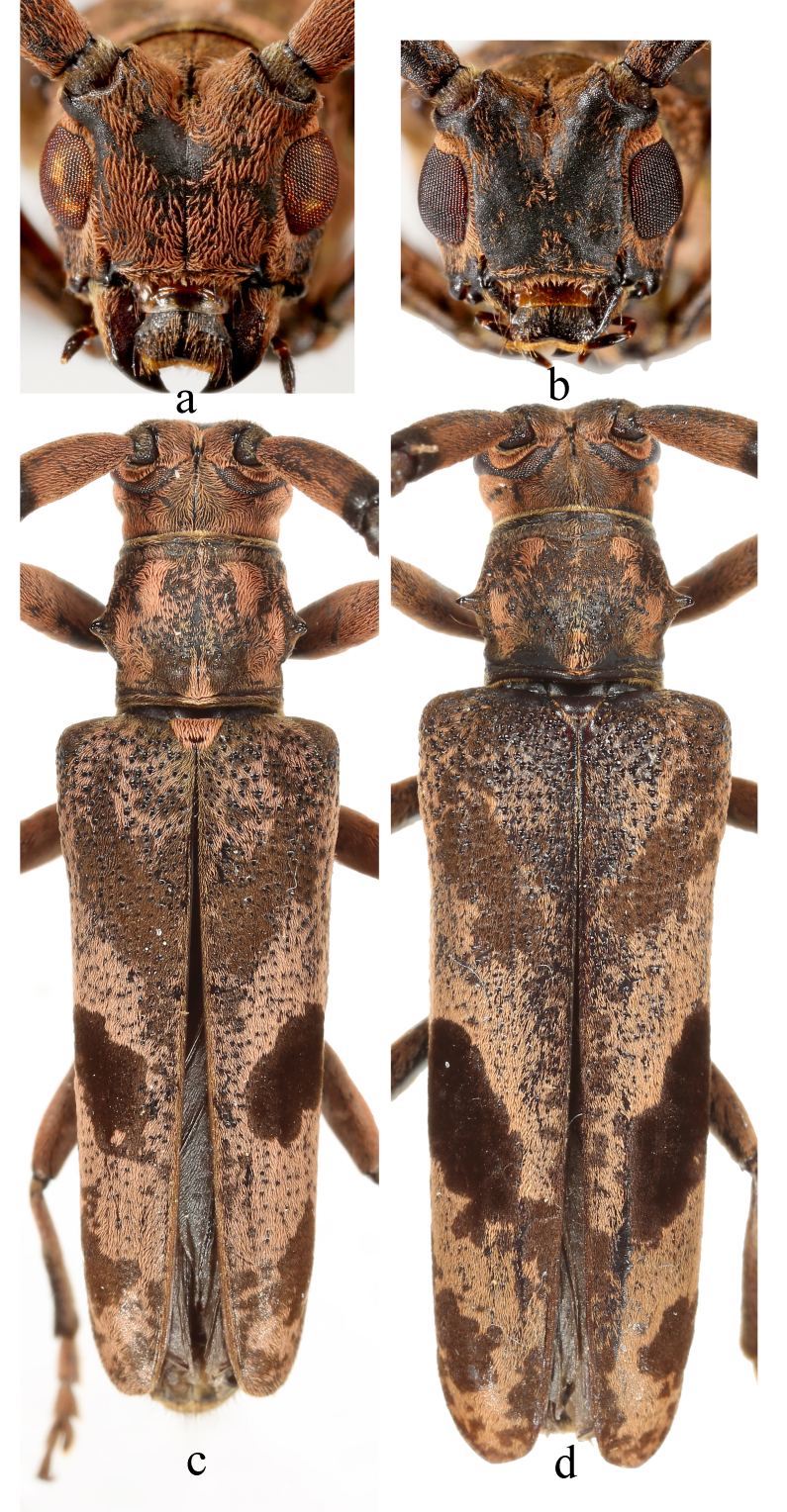
Habitus of *Uraechaangusta* (Pascoe, 1856). **a, c** male; **b, d** female; **a, b** head, front view; **c, d** dorsal view. Not to scale.

**Figure 4.**
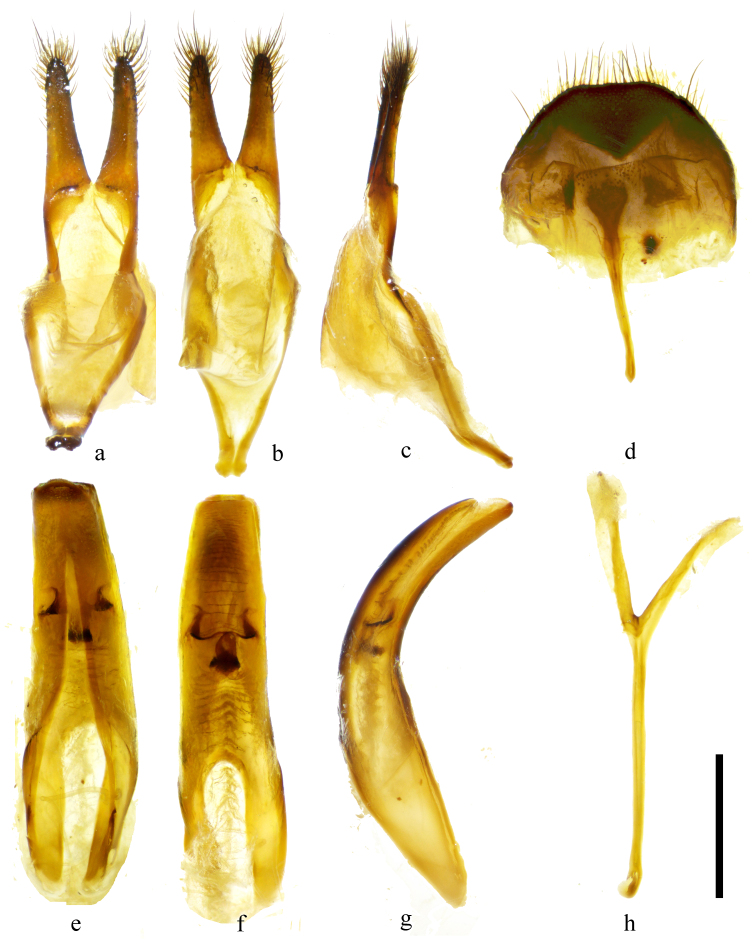
Male terminalia of *Uraechaangusta* (Pascoe, 1856). **a–c** parameres; **d** tergite VIII and sternites VIII; **e–g** median lobe; **h** spiculum gastrale (**a, d, e, h** ventral view; **b, f** dorsal view; **c, g** lateral view). Scale bar 0.5 mm.

*Uraechanigromaculata* sp. n. can be distinguished from its close relatives by the unique large semicircular dark marking behind the middle part of the elytron and the absence of an apical marking on the elytron. *Uraechaangusta* (Pascoe, 1856), a widespread species, which shows great morphological variability, is the closest relative of *U.nigromaculata* sp. n. in terms of morphology. The five longitudinal yellow stripes and density of granules on the pronotum and the size of the V-shaped dark marking and density of granules on the base of elytra are variable amongst individuals for both species. However, characteristics of body colouration and the markings behind the middle of elytra are fairly consistent within each species and different between these two species. The body colouration of *U.nigromaculata* sp. n. is generally lighter than that of *U.angusta*. The middle elytral marking in *U.angusta* is an oblique stripe and there is an irregular-shaped apical elytral marking, even light in colour in some individuals (Fig. 3c, d). For comparison, the middle elytral marking in *U.nigromaculata* sp. n. is a semicircular-shaped marking and there are no markings on the apical of elytra (Fig. 1a, b, i, j). The male terminalia of *U.angusta* are also similar to the male terminalia of *U.nigromaculata* sp. n. However, there are characteristic differences between the two species: (1) the lateral lobe is constricted from the base to basal 1/3, then gradually tapering to apex, with sparse, mostly short setae and only several long setae in *U.nigromaculata* sp. n. (Fig. 2a–c). In contrast, the lateral lobe gradually constricts from base to apex, with dense short and long setae at about apical 1/2 in *U.angusta* (Fig. 4a–c); (2) The basal half of median lobe is relatively broad in *U.nigromaculata* sp. n. (Fig. 2e–g), while it is narrow in *U.angusta* (Fig. 4e–g); (3) tergite VIII is less arcuated with a subtrapezoidal shape and the apex of the spiculum relictum is not widened in *U.nigromaculata* sp. n. (Fig. 2d), while tergite VIII is rounded and the apex of the spiculum relictum is widened in *U.angusta* (Fig. 4d).

#### Etymology

The specific name refers to the large dark marking bechind the middle of each elytron.

#### Taxon discussion

There are a number of intraspecific variations in the new species, especially in the pubescence pattern on the pronotum and elytra. Not all individuals show conspicuous light orange pubescence stripes present on the pronotum (Fig. 1k). In some individuals, the basal elytral patch obliquely reaches suture at about 2/5 of elytral length (Fig. 1i), while in others (Fig. 1a, b, j), it nearly reaches half of the elytral length. The edge of the large elytral patch behind the middle of elytra is incomplete and the inner margin is not rounded in some individuals (Fig. 1j). The apex of elytra is either subtruncate (Fig. 1a, b) or rounded (Fig. 1i, j).

### Uraecha angusta

(Pascoe, 1856)

DAAE5D7A-3186-5721-B87D-52C1106F1D18

#### Materials

- **Type status:** Other material. **Occurrence:** recordedBy: Shulin Yang and Run Shi; individualCount: 1; sex: male; lifeStage: adult; occurrenceID: F5436881-B81C-5591-81B7-93937ACC1A96; **Taxon:** scientificName: *Uraechaangusta*; **Location:** country: China; stateProvince: Guizhou; county: Xishui; locality: Wangxiantai, Sanchahe; verbatimCoordinates: 106°24.307’ E, 28°33.531’N; **Identification:** dateIdentified: 2022; **Event:** samplingProtocol: flight interception trap; year: 2022; month: 7; day: 21
- **Type status:** Other material. **Occurrence:** recordedBy: Shulin Yang and Run Shi; individualCount: 1; sex: female; lifeStage: adult; occurrenceID: AAE9A278-03E3-5C3E-81D2-D7D2C7C94759; **Taxon:** scientificName: *Uraechaangusta*; **Location:** country: China; stateProvince: Guizhou; county: Xishui; locality: Wangxiantai, Sanchahe; verbatimCoordinates: 106°24.192’ E, 28°33.766’N; **Identification:** dateIdentified: 2022; **Event:** samplingProtocol: flight interception trap; year: 2022; month: 10; day: 17

#### Taxon discussion

*Uraechaangusta* (Pascoe, 1856) is also a species that presents intraspecific variability (Lin and Ge 2017). It is closer to its relatives with oblique elytral markings, *Uraechachinensis* (Breuning 1935) and *Uraechaobliquefasciata* Chiang, 1951.

## Supplementary Material

XML Treatment for Uraecha nigromaculata

XML Treatment for Uraecha angusta
